# Supplementary material for: Predicting performance in 4 x 200-m freestyle swimming relay events
Source: PLoS One. 2021 Jul 15;16(7):e0254538. doi: 10.1371/journal.pone.0254538 (PMC8282077; doi:10.1371/journal.pone.0254538)
Supplement: S1 Fig — Note that the residuals are mostly normal and randomly distributed. Although there is a little deviation from normality in the right tail of the distribution (ignoring the outlier), there are relatively few data points here and these slow swim times are not as relevant in the context of predicting medalling performances. (DOCX) [file pone.0254538.s001.docx]

**
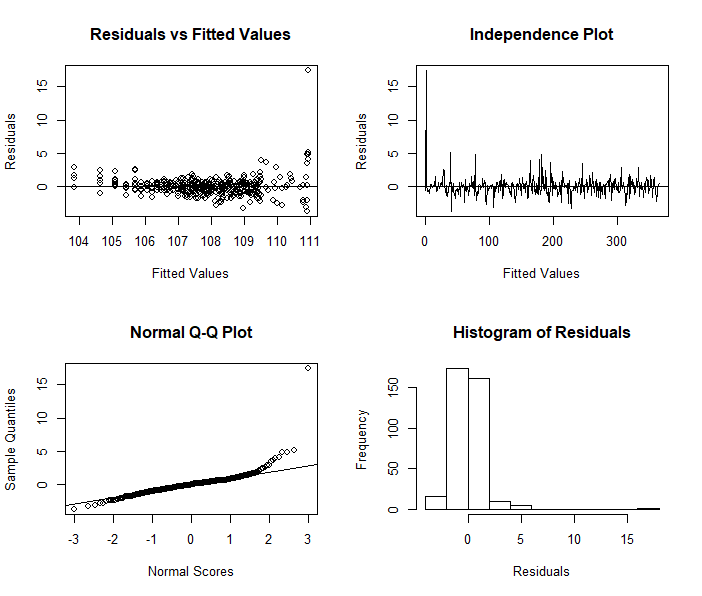
**

S1 Fig: Residual and normality plot for the linear regression model reported in Results. Note that the residuals are mostly normal and randomly distributed. Although there is a little deviation from normality in the right tail of the distribution (ignoring the outlier), there are relatively few data points here and these slow swim times are not as relevant in the context of predicting medalling performances.
