# Supplementary material for: Predicting performance in 4 x 200-m freestyle swimming relay events
Source: PLoS One. 2021 Jul 15;16(7):e0254538. doi: 10.1371/journal.pone.0254538 (PMC8282077; doi:10.1371/journal.pone.0254538)
Supplement: Attachment — Submitted filename: Response to Reviewers.docx [file pone.0254538.s004.docx]

Dear Prof. Filho

Thank you to you and the reviewers for your feedback. Please see below our responses highlighted in yellow and excerpts from the updated paper highlighted in gray. Please note that the line numbers in the response refer to Manuscript.docx.

Thanks

Paul

On Tue, 15 Jun 2021 at 18:42, PLOS ONE <em@editorialmanager.com> wrote:

PONE-D-21-03614R1
Predicting performance in 4 x 200-m freestyle swimming relay events
PLOS ONE

Dear Dr. Wu,

Thank you for submitting your manuscript to PLOS ONE. After careful consideration, we feel that it has merit but does not fully meet PLOS ONE’s publication criteria as it currently stands. Therefore, we invite you to submit a revised version of the manuscript that addresses the points raised during the review process.

Please submit your revised manuscript by Jul 30 2021 11:59PM. If you will need more time than this to complete your revisions, please reply to this message or contact the journal office at [plosone@plos.org](mailto:plosone@plos.org). When you're ready to submit your revision, log on to [https://www.editorialmanager.com/pone/](https://urldefense.com/v3/__https:/www.editorialmanager.com/pone/__;!!NVzLfOphnbDXSw!RY6xkgmiNHUDmVKLXaTqH1BFhVMP-aAcRWoWkmUiNhP0WPTqeH8icdTaJ81YZWdH$) and select the 'Submissions Needing Revision' folder to locate your manuscript file.

Please include the following items when submitting your revised manuscript:

- A rebuttal letter that responds to each point raised by the academic editor and reviewer(s). You should upload this letter as a separate file labeled 'Response to Reviewers'.
- A marked-up copy of your manuscript that highlights changes made to the original version. You should upload this as a separate file labeled 'Revised Manuscript with Track Changes'.
- An unmarked version of your revised paper without tracked changes. You should upload this as a separate file labeled 'Manuscript'.

If you would like to make changes to your financial disclosure, please include your updated statement in your cover letter. Guidelines for resubmitting your figure files are available below the reviewer comments at the end of this letter.

If applicable, we recommend that you deposit your laboratory protocols in [protocols.io](http://protocols.io) to enhance the reproducibility of your results. Protocols.io assigns your protocol its own identifier (DOI) so that it can be cited independently in the future. For instructions see: [http://journals.plos.org/plosone/s/submission-guidelines#loc-laboratory-protocols](https://urldefense.com/v3/__http:/journals.plos.org/plosone/s/submission-guidelines*loc-laboratory-protocols__;Iw!!NVzLfOphnbDXSw!RY6xkgmiNHUDmVKLXaTqH1BFhVMP-aAcRWoWkmUiNhP0WPTqeH8icdTaJzIAN9p6$). Additionally, PLOS ONE offers an option for publishing peer-reviewed Lab Protocol articles, which describe protocols hosted on [protocols.io](http://protocols.io). Read more information on sharing protocols at [https://plos.org/protocols?utm_medium=editorial-email&utm_source=authorletters&utm_campaign=protocols](https://urldefense.com/v3/__https:/plos.org/protocols?utm_medium=editorial-email&utm_source=authorletters&utm_campaign=protocols__;!!NVzLfOphnbDXSw!RY6xkgmiNHUDmVKLXaTqH1BFhVMP-aAcRWoWkmUiNhP0WPTqeH8icdTaJ-0HLl5o$).

We look forward to receiving your revised manuscript.

Kind regards,

Dalton Müller Pessôa Filho, Ph.D.
Academic Editor
PLOS ONE

Journal Requirements:

Please review your reference list to ensure that it is complete and correct. If you have cited papers that have been retracted, please include the rationale for doing so in the manuscript text, or remove these references and replace them with relevant current references. Any changes to the reference list should be mentioned in the rebuttal letter that accompanies your revised manuscript. If you need to cite a retracted article, indicate the article’s retracted status in the References list and also include a citation and full reference for the retraction notice.

Additional Editor Comments (if provided):

The reviewers congratulate the authors on the improvement of this manuscript after the first round of revision. However, Reviewer #2 has addressed two new comments to the authors. Therefore, the authors need to provide the responses to these other comments before the final decision.

[Note: HTML markup is below. Please do not edit.]

Reviewers' comments:

Reviewer's Responses to Questions

**Comments to the Author**

1. If the authors have adequately addressed your comments raised in a previous round of review and you feel that this manuscript is now acceptable for publication, you may indicate that here to bypass the “Comments to the Author” section, enter your conflict of interest statement in the “Confidential to Editor” section, and submit your "Accept" recommendation.

Reviewer #1: All comments have been addressed

Reviewer #2: All comments have been addressed

2. Is the manuscript technically sound, and do the data support the conclusions?

The manuscript must describe a technically sound piece of scientific research with data that supports the conclusions. Experiments must have been conducted rigorously, with appropriate controls, replication, and sample sizes. The conclusions must be drawn appropriately based on the data presented.

Reviewer #1: (No Response)

Reviewer #2: Yes

3. Has the statistical analysis been performed appropriately and rigorously?

Reviewer #1: (No Response)

Reviewer #2: Yes

4. Have the authors made all data underlying the findings in their manuscript fully available?

The [PLOS Data policy](https://urldefense.com/v3/__http:/www.plosone.org/static/policies.action*sharing__;Iw!!NVzLfOphnbDXSw!RY6xkgmiNHUDmVKLXaTqH1BFhVMP-aAcRWoWkmUiNhP0WPTqeH8icdTaJwtkg66v$) requires authors to make all data underlying the findings described in their manuscript fully available without restriction, with rare exception (please refer to the Data Availability Statement in the manuscript PDF file). The data should be provided as part of the manuscript or its supporting information, or deposited to a public repository. For example, in addition to summary statistics, the data points behind means, medians and variance measures should be available. If there are restrictions on publicly sharing data—e.g. participant privacy or use of data from a third party—those must be specified.

Reviewer #1: (No Response)

Reviewer #2: Yes

5. Is the manuscript presented in an intelligible fashion and written in standard English?

PLOS ONE does not copyedit accepted manuscripts, so the language in submitted articles must be clear, correct, and unambiguous. Any typographical or grammatical errors should be corrected at revision, so please note any specific errors here.

Reviewer #1: (No Response)

Reviewer #2: Yes

6. Review Comments to the Author

Please use the space provided to explain your answers to the questions above. You may also include additional comments for the author, including concerns about dual publication, research ethics, or publication ethics. (Please upload your review as an attachment if it exceeds 20,000 characters)

Reviewer #1: Authors have adequately addressed the comments raised in a previous round of review. However, it was difficult for the present reviewer to find the authors’ responses within the text. Authors should indicate their responses by bullet points or “response”.

Reviewer #2: General comments

Thank you for accepting the suggestions. I would like to suggest two more things:

The objective should coincide with the Abstract (lines 33-36) and the Introduction (lines 104-107). To predict and to enhance are not the same.

We have made the Introduction consistent with the Abstract (line 105):

Therefore, the aim of this study was to predict and better understand contextual factors contributing to relay team performance in light of individual swimmer performance, and develop predictive models to analyse the relationship between a team’s finishing position and these factors for the 4x200-m swimming freestyle relay.

Line 101-104. Please, clarify more why the study was done in 4 x 200-m swimming freestyle relay events

We have clarified as follows (line 101):

With each swimmer required to complete 4 x 50 m laps the event requires well-developed speed-endurance, technical skills in starts, turns and finishes, and the element of pacing and sufficient data to model pacing effects [9]. This complexity makes the 4 x 200-m freestyle ideal as a starting point for developing and testing predictive and analytical tools.

7. PLOS authors have the option to publish the peer review history of their article ([what does this mean?](https://urldefense.com/v3/__https:/journals.plos.org/plosone/s/editorial-and-peer-review-process*loc-peer-review-history__;Iw!!NVzLfOphnbDXSw!RY6xkgmiNHUDmVKLXaTqH1BFhVMP-aAcRWoWkmUiNhP0WPTqeH8icdTaJ4BAl1Np$)). If published, this will include your full peer review and any attached files.


If you choose “no”, your identity will remain anonymous but your review may still be made public.


**Do you want your identity to be public for this peer review?** For information about this choice, including consent withdrawal, please see our [Privacy Policy](https://urldefense.com/v3/__https:/www.plos.org/privacy-policy__;!!NVzLfOphnbDXSw!RY6xkgmiNHUDmVKLXaTqH1BFhVMP-aAcRWoWkmUiNhP0WPTqeH8icdTaJ0aIGCsE$).

Reviewer #1: **Yes:** Santi Veiga

Reviewer #2: No

[NOTE: If reviewer comments were submitted as an attachment file, they will be attached to this email and accessible via the submission site. Please log into your account, locate the manuscript record, and check for the action link "View Attachments". If this link does not appear, there are no attachment files.]

While revising your submission, please upload your figure files to the Preflight Analysis and Conversion Engine (PACE) digital diagnostic tool, [https://pacev2.apexcovantage.com/](https://urldefense.com/v3/__https:/pacev2.apexcovantage.com/__;!!NVzLfOphnbDXSw!RY6xkgmiNHUDmVKLXaTqH1BFhVMP-aAcRWoWkmUiNhP0WPTqeH8icdTaJ2uBussZ$). PACE helps ensure that figures meet PLOS requirements. To use PACE, you must first register as a user. Registration is free. Then, login and navigate to the UPLOAD tab, where you will find detailed instructions on how to use the tool. If you encounter any issues or have any questions when using PACE, please email PLOS at [figures@plos.org](mailto:figures@plos.org). Please note that Supporting Information files do not need this step.

*In compliance with data protection regulations, you may request that we remove your personal registration details at any time.* [*(Remove my information/details)*](https://urldefense.com/v3/__https:/www.editorialmanager.com/pone/login.asp?a=r__;!!NVzLfOphnbDXSw!RY6xkgmiNHUDmVKLXaTqH1BFhVMP-aAcRWoWkmUiNhP0WPTqeH8icdTaJ3YEbtyl$)*. Please contact the publication office if you have any questions.*

--

**Dr Paul Wu** |  Senior Lecturer, Associate Investigator

ARC Centre of Excellence for Mathematical & Statistical Frontiers (ACEMS)

Centre for Data Science (CDS)

Faculty of Science

Queensland University of Technology

**ph** +61 3138 9828 |  **email** [p.wu@qut.edu.au](mailto:XXXXXXXXXX@qut.edu.au)

CRICOS No 00213J  |  ABN: 83 791 724 622
